# Comparison of a Mobile Health Electronic Visual Analog Scale App With a Traditional Paper Visual Analog Scale for Pain Evaluation: Cross-Sectional Observational Study

**DOI:** 10.2196/18284

**Published:** 2020-09-17

**Authors:** Alexandra Turnbull, Dean Sculley, Carles Escalona-Marfil, Lluís Riu-Gispert, Jorge Ruiz-Moreno, Xavier Gironès, Andrea Coda

**Affiliations:** 1 School of Health Sciences Faculty of Health and Medicine The University of Newcastle Ourimbah Australia; 2 School of Biomedical Sciences and Pharmacy Faculty of Health and Medicine The University of Newcastle Ourimbah Australia; 3 Facultat de Ciències de la Salut de Manresa Universitat de Vic-Universitat Central de Catalunya Manresa Spain; 4 Department of Physical Therapy Escola Universertària de la Salut i l’Esport (EUSES) University of Girona Salt, Girona Spain; 5 MIXESTAT SL Barcelona Spain; 6 Priority Research Centre Health Behaviour Hunter Medical Research Institute Newcastle Australia

**Keywords:** pain, mobile app, mHealth, digital health, electronic visual analog scale, visual analog scale, symptom, eHealth, reliability

## Abstract

**Background:**

Accurate quantification of pain in a clinical setting is vital. The use of an electronic pain scale enables data to be collected, analyzed, and utilized much faster compared with traditional paper-based scales. The advancement of smart technology in pediatric and adult pain evaluation may offer opportunities to introduce easy-to-use and reliable pain assessment methods within different clinical settings. If promptly introduced within different pediatric and adult pain clinic services, validated and easily accessible mobile health pain apps may lead to early pain detection, promoting improvement in patient’s quality of life and leading to potentially less time off from school or work.

**Objective:**

This cross-sectional observational study aimed to investigate the interchangeability of an electronic visual analog scale (eVAS) app with a traditional paper visual analog scale (pVAS) among Australian children, adolescents, and adults for pain evaluation.

**Methods:**

Healthy participants (age range 10-75 years) were recruited from a sporting club and a secondary school in Melbourne (Australia). The data collection process involved application of pressure (8.5 kg/cm^2^) from a Wagner Force Dial FDK 20 to the midpoint of the thumb. The pressure was applied twice with a 5-minute interval. At each pressure application, participants were asked to randomly record their pain perception using the “eVAS” accessible via the “Interactive Clinics” app and the traditional pVAS. Statistical analysis was conducted to determine intermethod and intramethod reliabilities.

**Results:**

Overall, 109 healthy participants were recruited. Adults (mean age 42.43 years, SD 14.50 years) had excellent reliability, with an intraclass correlation coefficient (ICC) of 0.94 (95% CI 0.91-0.96). Children and adolescents (mean age 13.91 years, SD 2.89 years) had moderate-to-good intermethod and intramethod reliabilities, with an ICC of 0.80 (95% CI 0.70-0.87) and average ICC of 0.80 (95% CI 0.69-0.87), respectively.

**Conclusions:**

The eVAS app appears to be interchangeable compared with the traditional pVAS among children, adolescents, and adults. This pain evaluation method may offer new opportunities to introduce user-friendly and validated pain assessment apps for patients, clinicians, and allied health professionals.

## Introduction

Pain is a complex and multifactorial phenomenon that can negatively impact a patient’s health-related quality of life [1]. Pain outcome measures are commonly used to assess the severity of symptoms in children, adolescents, and adults [2]. Traditionally, symptom progression has been recorded using the visual analog scale (VAS), Wong Baker scale, numeric rating scale, verbal rating scale, and faces pain scale-revised [3-6]. These tools have been extensively validated as appropriate measures for assessing pain and are commonly used daily by allied health professionals (AHPs).

Evidence suggests there are limitations associated with the more traditional paper pain outcome measures that are still commonly used in various clinical settings [7]. Drawn face scales may result in incorrect recordings if a child experiences difficulty in distinguishing between the feeling of pain and the emotional state, and smiling faces could result in overestimation of pain intensity [4]. These limitations are mostly based on paper pain scales being cumbersome, occasionally complex to use, and at risk of possible practitioner error [8].

The continuous growth of mobile health (mHealth) offers unparalleled opportunities to address issues related to health systems and accessing accurate, reliable, and frequent health data [9,10]. The introduction of smart technology in pediatric and adult pain evaluation may offer opportunities to implement tailored pain assessments within different clinical settings. Recently, novel technologies have emerged that utilize smart devices to improve the existing traditional pain outcome measures [11-16]. Few of these novel technologies have been examined for reliability and validity among children, adolescents, and adults, with most employing small sample sizes. There is growing evidence to suggest that electronic pain outcome measures are interchangeable with existing traditional pain outcome measures, but more mHealth and eHealth research is needed to test the validity and reliability of the electronic VAS (eVAS) among children and adolescents [17]. By evaluating the validity of mHealth and eHealth interventions available to patients and clinicians, we can equip AHPs with a more effective tool to measure symptom progression. This study adheres to the standards of digital health interventions set by the World Health Organization (WHO) in 2016 [18] and the Australian Government Health Authorities’ guidelines with regard to how to validate new digital mHealth systems for the benefit of patients, clinicians, and the community [19]. These include monitoring and evaluating linear stages of development from prototype through to pilot studies showing efficacy, demonstration of effectiveness, scaling up, and integration into the clinical environment [18]. The traditional paper VAS (pVAS) was used owing to its well-established validity across all age groups [20]. This cross-sectional observational study aimed to investigate the interchangeability between an eVAS app and a traditional pVAS among children, adolescents, and adults. This may validate the use of the eVAS in a clinical setting, enhancing the collection, analysis, and dissemination of data in line with the eHealth and mHealth pathways envisaged by health authorities worldwide.

## Methods

### Recruitment

Healthy participants were recruited through a convenient sample of participants in Melbourne (Victoria, Australia) from John Monash Science School (Clayton) and KBH Brumbies Hockey Club (Mont Albert North). English speaking children and adolescents (age range 10-18 years) and adults (age range 18-75 years) were eligible to participate in the study. A mean age of 9.8 years appears to be suitable to evaluate the concept of experienced pain [3]; therefore, this finding was utilized to inform the age group for this mHealth trial, despite the possibility to reliably use the traditional pVAS from the age of 7 years [20]. Participants were excluded if they were diagnosed with neurological disorders, were receiving medication that would alter pain perception or threshold, and had severe visual impairments that may prevent viewing the pVAS and eVAS.

Prior to consent approval, a participant information sheet was supplied to potential participants, and they were made aware of the procedure and the time commitments required to take part in the study. The participant information sheet was adapted according to age (adults, and children and adolescents) including more visual aids in the children’s version. Ethical approval was obtained from the University of Newcastle Human Research Ethics Committee (Dev-005638). Approval was also sought from and granted by the Victorian Schools and the Department of Education (Victoria) (2018_00373). Participants’ gender and age were recorded and their identities were completely anonymized, with a unique ID assigned to each participant.

### Measuring Instruments

**Figure 1:**
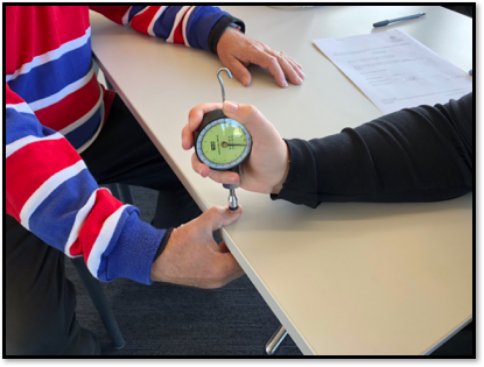
Wagner Force Dial pressure applied on the participant’s thumb.

In order to apply a standardized pressure to the participant’s thumb, a Wagner Force Dial FDK 20 with a 1 cm^2^ circular rubber end was adopted for each data collection, and the same data collector (AT) completed all measurements. The setup of the collection included a simple table and chair. The participant sat on the chair with the thumb on the edge of the table and other fingers underneath. A vertical pressure (8.5 kg/cm^2^) using the Force Dial was applied to the midpoint of the thumb for 3 seconds (Figure 1).

**Figure 2:**
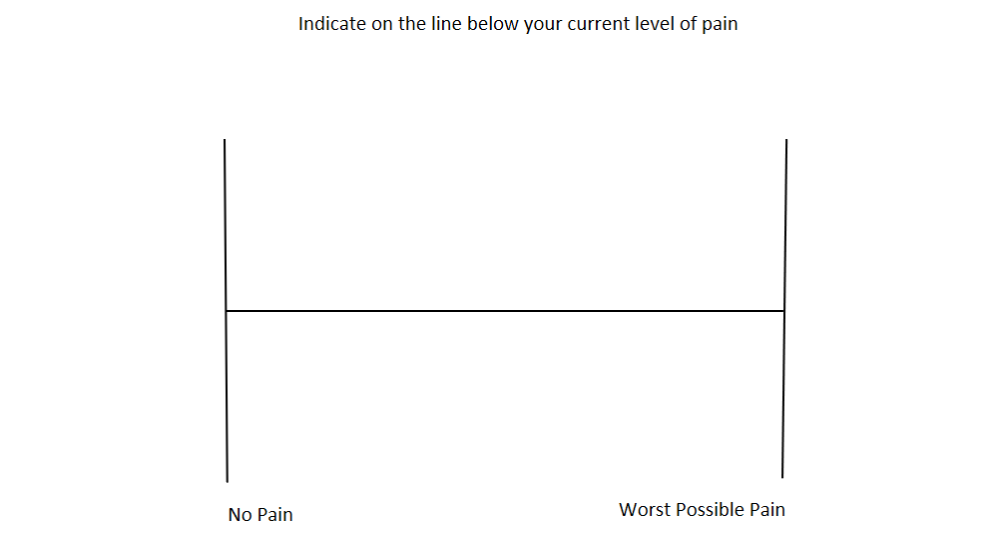
Standard paper visual analog scale that was printed on a white background A4-sized paper.

**Figure 3:**
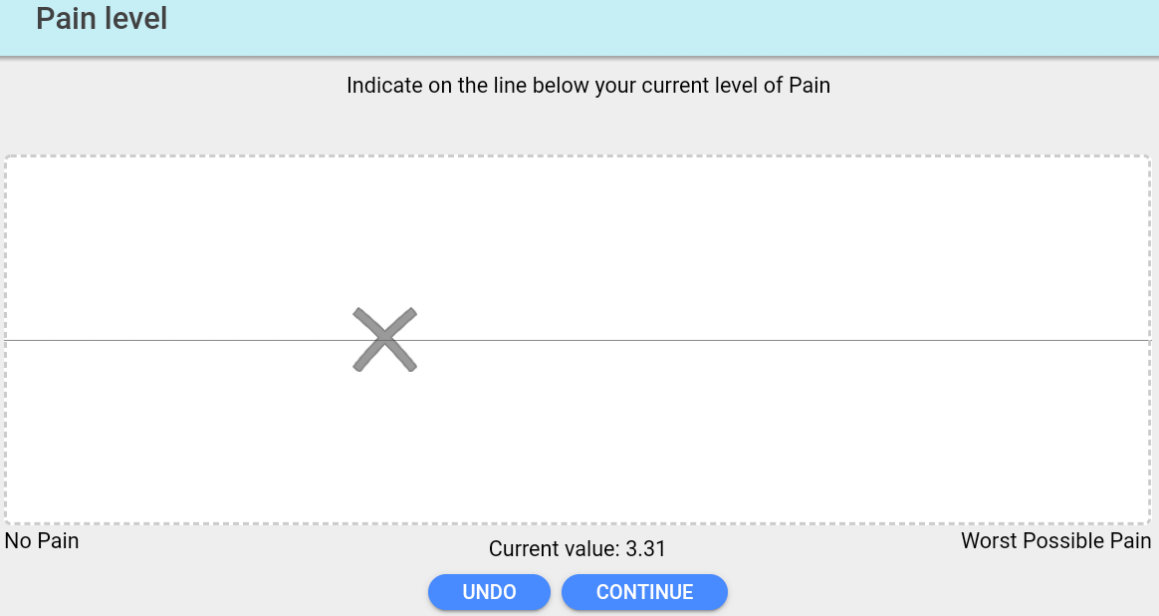
The electronic visual analog scale accessible via the Interactive Clinics app developed by BitGenoma Ltd, Spain.

The chosen amount of pressure (kg/cm^2^) applied to the participant’s thumb was previously successfully used by Escalona-Marfil et al and provides light enough pressure to mimic symptoms of mild pain while not being too extensive as to generate stronger pain or skin damage [17,21]. After application of pressure, the participant was asked to randomly complete either the pVAS (Figure 2) or eVAS (Figure 3).

The order of measurement using the pVAS and eVAS was block randomized (groups of 10). Sequential allocation was achieved using a freely available random number generator [22]. Allocation concealment for the eVAS and pVAS sequence was achieved by using sequentially numbered, opaque, and sealed envelopes. Both sequential generation and allocation concealment were conducted by an independent research team member (AC) who was not involved during the data collection process and did not have any prior or ongoing contact with enrolled participants.

With regard to the pVAS, participants were asked to draw a vertical line that corresponded with their symptom level, with the left side corresponding to “no pain” and the right side corresponding to “the worst pain imaginable” [21]. The traditional pVAS was a 12 × 7.5-cm white paper sheet with a 10-cm horizontal line drawn and two vertical 6-cm lines drawn either side. The eVAS was accessible via the “Interactive Clinics” app, and all recordings were conducted on a 7-inch (17.8 cm) Samsung Galaxy Tab 3 with Android operating system (v5.1.1), displaying a 13.5-cm straight horizontal line on a white background. When using the eVAS, the tablet was placed horizontally on the table at all times and each participant was asked to place one finger on the line on the screen [17]. In order to prevent bias, the data collector (AT) wiped the tablet screen completely between measurements to prevent the participant from being able to see the position of the fingerprint previously placed on the tablet’s screen.

After a period of 5 minutes, the sequence of data collection was reversed for each individual. Data collection only took approximately 10-15 minutes in total for each participant, and no follow-up was required. The use of standardized pressure application with 1-minute intervals was reliable for absolute pressure thresholds in multiple studies [23,24]. Additionally, simple pressure algometry is a repeatable measure of pain threshold [25,26]. A 5-minute interval was introduced to reduce possible risk of reacting pre-emptively to stimuli and to prevent temporal summation that could have impacted the quality of the assessment. For logistical reasons, the data collector (AT) was not blinded to this process. However, as soon as the values on the pVAS and eVAS were recorded, the tablet or the paper was immediately withheld from the participant in order to prevent any possible modifications to the data entered by the participant. The results gathered from the pVAS were extrapolated (by AT) using a standard plastic ruler, whereas the eVAS value was automatically calculated by the software.

### Statistical Analysis

For a 5% two-sided *t* test with α=.05 and 80% power in an observational cross-sectional study with one intervention observation and a moderate effect size, it was estimated that a total of 100 subjects would be required [27,28]. The study was overpowered to an estimated 110 subjects (55 aged 10-18 years and 55 aged 18-75 years) to allow for a 9% dropout rate during the data collection period. Summary statistics for eVAS and pVAS results were calculated by splitting the measurement and method. Two approaches have been used to evaluate the agreement of the two methods (intermethod and intramethod reliabilities by means of intraclass correlation coefficients). STATA 15 (StataCorp LLC) was utilized for statistical analysis [29]. The independent statistician was blinded to the eVAS and pVAS allocation concealment and to the participant identity.

### Intermethod and Intramethod Agreement Analysis

A mixed factorial model was employed to derive two intraclass correlation coefficients according to Shrout-Fleiss reliability fixed set [29,30]. One coefficient was a measure of intermethod reliability (ρ) estimated by the intraclass correlation coefficient (ICC). This coefficient was defined as the correlation between VAS values from different methods in the same subject and same replication. The other intraclass coefficient (γ) estimated by the average ICC (ICCa) was used as a measure of intramethod reliability. This was defined as the correlation between VAS values in the same method and same subject. A two-way balanced mixed analysis of variance model without interaction, a random subject effect, and a fixed method effect was fitted to estimate ICCs. The mean of squares for subjects, subject-method interactions, and errors from components of variance were also calculated [31]. Statistical inference of the ICCs was performed with CIs. In order to improve reliability coefficients, 95% CIs were calculated from the estimated sum of squares. For both intermethod reliability and intramethod reliability, the ICCs were higher than 0.8. In order to specify the precision of the estimated ICC, the length of the 95% CI was expressed as a function of the ICC value. Given that it was not possible to increase the number of methods to evaluate the VAS, the number of subjects was increased. In children and adolescents, with 94 ratings per method (47 subjects with two replicates per subject) and an anticipated ICC value of at least 0.8, an acceptable length for the 95% CI will be less than or equal to 0.2. In adults, with 124 ratings per method (62 subjects with two replicates per subject) and an anticipated ICC value of at least 0.8, an acceptable length for the 95% CI will be less than or equal to 0.1. Good agreement among methods was evaluated by plotting both methods against subjects and performing a Wilcoxon rank-sum test. According to Portney and Watkins, ICC values are classified as follows: ICC < 0.5, poor; 0.5 ≤ ICC ≤ 0.75, moderate; 0.75 < ICC ≤ 0.9, good; and ICC > 0.9, excellent [30].

## Results

### Participant Characteristics

A total of 109 participants were included in the study. The study population consisted of 47 children and adolescents (mean age 13.9 years, SD 2.89 years; range 10-18 years; 16 female and 31 male participants) and 62 adults (mean age 42.44 years, SD 14.50 years; range 19-73 years; 37 female and 25 male participants) (Table 1 and Table 2, respectively). No participants were lost to the analysis.

**Table 1:** Summary statistics (visual analog scale measurements) for children and adolescents.

Variable	First measure		Second measure	
	eVAS^a^	pVAS^b^	eVAS	pVAS
Number	47	47	47	47
Mean value	1.692553	1.657447	1.774681	1.642979
SD value	0.977331	1.039177	1.03974	1.000408
P50^c^	1.45	1.60	1.55	1.50
Minimum value	0.05	0.00	0.00	0.00
Maximum value	3.65	4.00	4.42	4.00

^a^eVAS: electronic visual analog scale.

^b^pVAS: paper visual analog scale.

^c^P50: middle estimate.

**Table 2:** Summary statistics (visual analog scale measurements) for adults.

Variable	First measure		Second measure	
	eVAS^a^	pVAS^b^	eVAS	pVAS
Number	62	62	62	62
Mean value	1.738387	1.690323	1.819839	1.759677
SD value	1.550611	1.571723	1.748486	1.743415
P50^c^	1.245	1.100	1.265	1.300
Minimum value	0.05	0.00	0.00	0.00
Maximum value	7.91	8.10	8.26	8.50

^a^eVAS: electronic visual analog scale.

^b^pVAS: paper visual analog scale.

^c^P50: middle estimate.

### Analysis of the eVAS and pVAS in Children, Adolescents, and Adults

**Figure 4:**
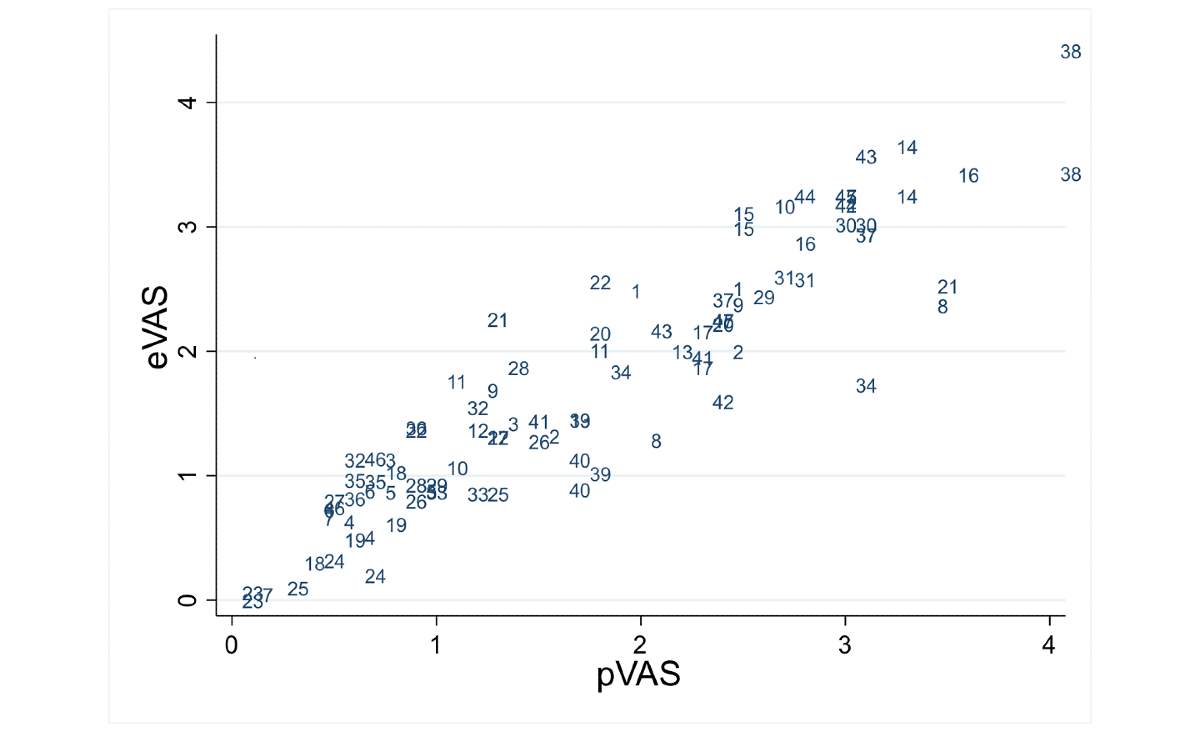
Scatter plot of the data in children and adolescents. Points are represented by subject number. eVAS: electronic visual analog scale; pVAS: paper visual analog scale.

**Figure 5:**
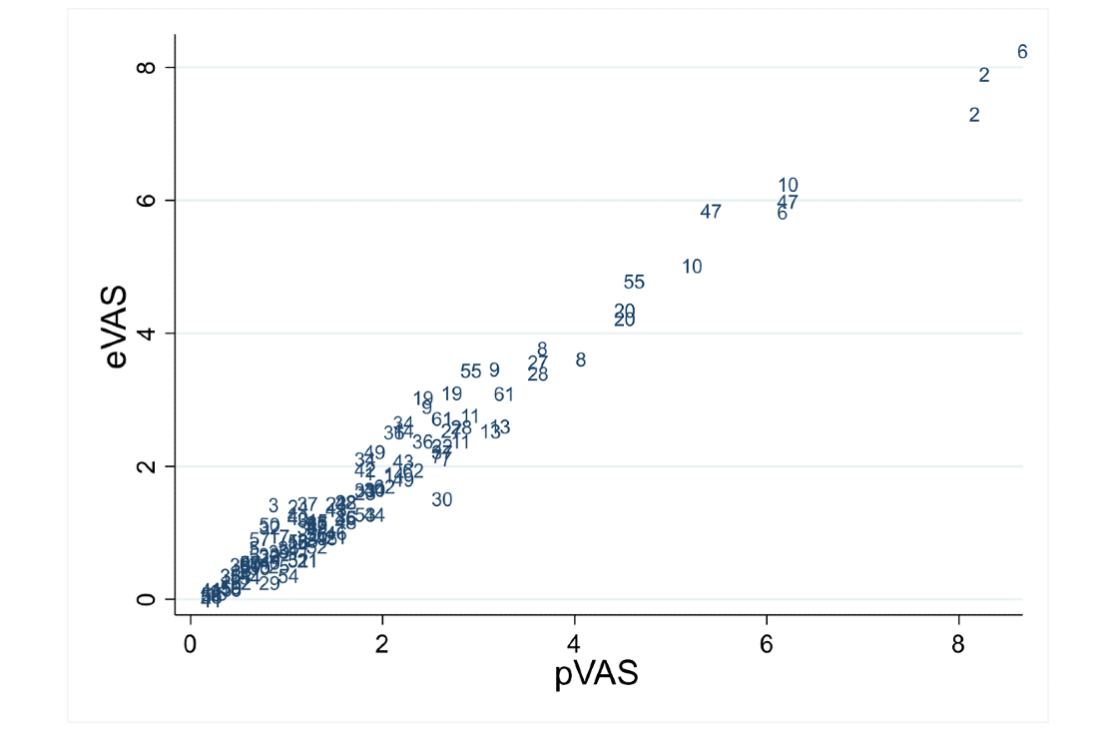
Scatter plot of the data in adults. Points are represented by subject number. eVAS: electronic visual analog scale; pVAS: paper visual analog scale.

Tables 1 and 2 show summary statistics for VAS measurements by measurement order and instrument (eVAS and pVAS) for the child and adolescent group and adult group, respectively. Differences between methods for median values ranged from 0.05 to 0.15 in children and adolescents and from 0.035 to 0.145 in adults. In Figures 4 and 5, the scatter plots for eVAS compared with pVAS are displayed for the child and adolescent group and the adult group, respectively, showing agreement between the two methods.

**Figure 6:**
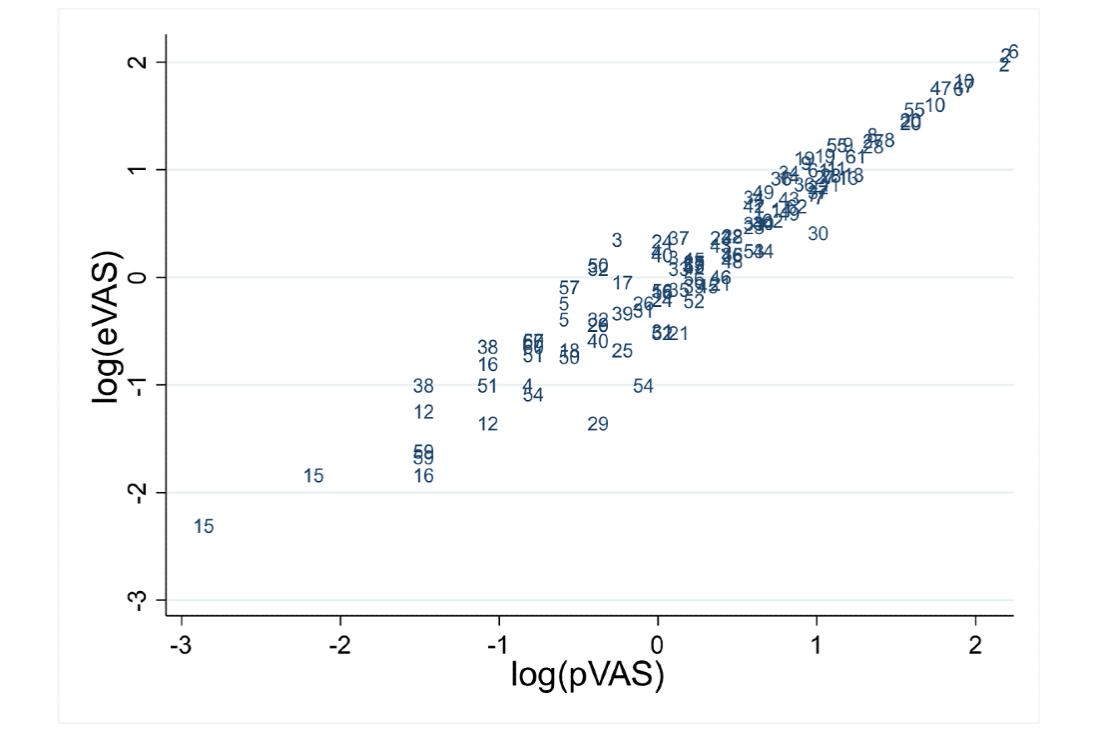
Scatter plot of the data (log) in adults. Points are represented by subject number. eVAS: electronic visual analog scale; pVAS: paper visual analog scale.

It is possible to observe in adults, the dispersion from values of VAS around 3, which is corrected when taking natural logarithms into account (Figure 6).

**Figure 7:**
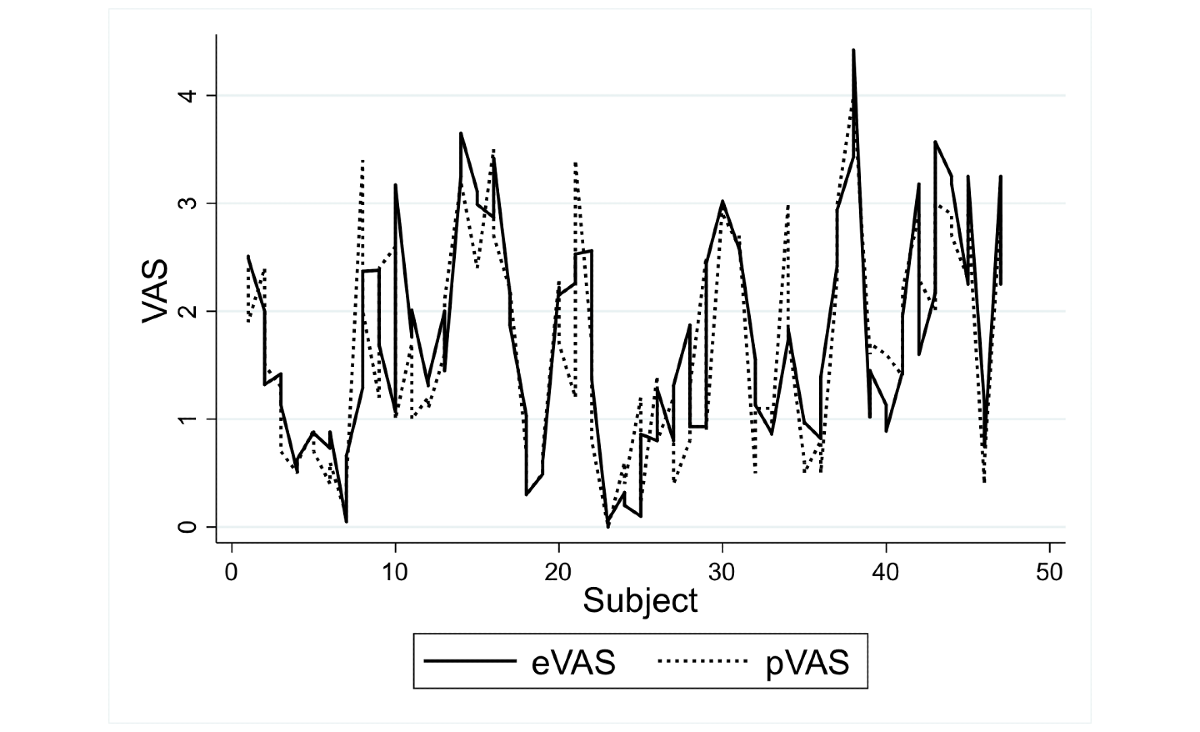
Rating data for the two methods in children and adolescents. eVAS: electronic visual analog scale; pVAS: paper visual analog scale.

**Figure 8:**
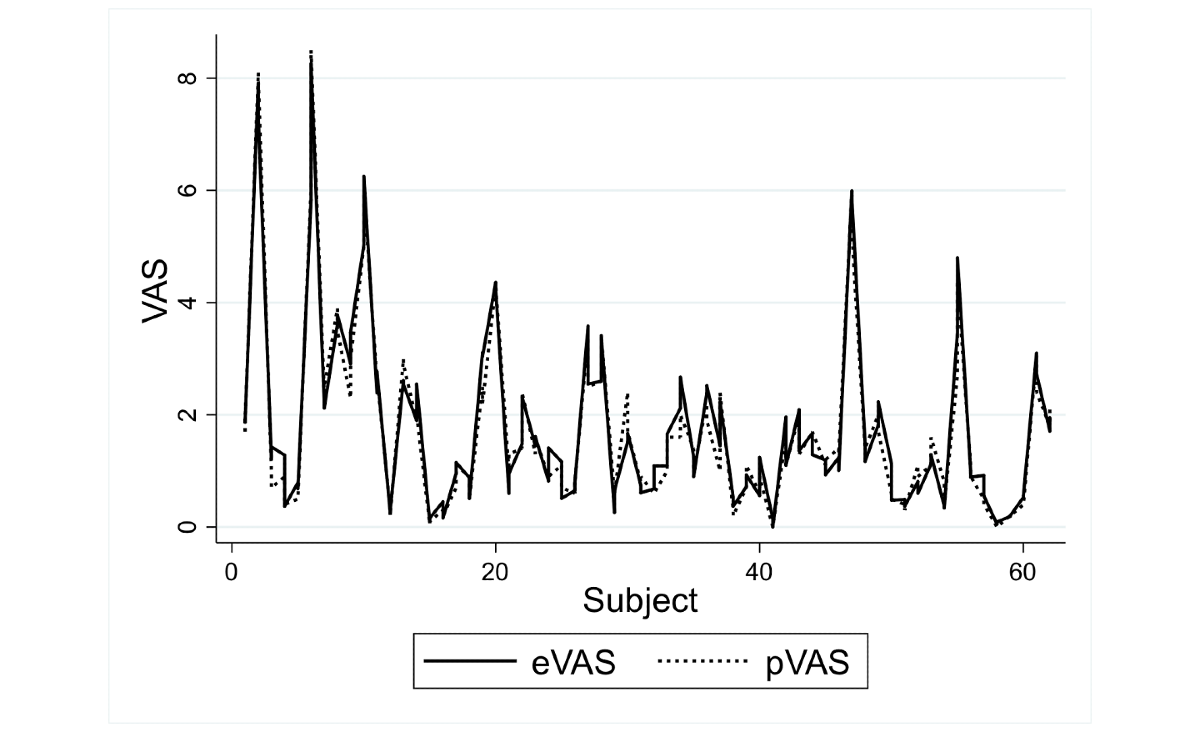
Rating data for the two methods in adults. eVAS: electronic visual analog scale; pVAS: paper visual analog scale.

Figures 7 and 8 show the agreement between the methods for children and adolescents, and adults, respectively.

The two-sample Wilcoxon rank-sum test for comparing methods was not significant (children and adolescents, *P*=.48; adults, *P*=.73). The normality of residuals of the model for the child and adolescent group and the adult group showed a centered distribution (children and adolescents: Shapiro-Francia test, *P*=.05 and Shapiro-Wilks test, *P*=.06; adults: Shapiro-Francia test, *P*=.13 and Shapiro-Wilks test, *P*=.21).

In children and adolescents, the intermethod reliability estimated by ICC reached the value of 0.80 (95% CI 0.70-0.87), indicating moderate-to-good reliability. The intramethod reliability estimated by ICCa reached the value of 0.80 (95% CI 0.69-0.87), indicating moderate-to-good reliability. For both coefficients, the length of the interval was less than 0.2.

In adults, the intermethod reliability estimated by ICC reached the value of 0.94 (95% CI 0.91-0.96), indicating excellent reliability. The intramethod reliability estimated by ICCa reached the value of 0.94 (95% CI 0.91-0.96), indicating excellent reliability [32]. For both coefficients, the length of the interval was 0.1 or less.

## Discussion

### Principal Findings

In the adult population, this study supported the interchangeability of the eVAS and pVAS owing to excellent intermethod and intramethod reliabilities as determined by an ICC value of 0.94 (95% CI 0.91-0.96) and ICCa value of 0.94 (95% CI 0.91-0.96), respectively. This supports previous findings by Bird et al who also reported excellent reliability in older adults (age range 65-85 years), using an apple iPad eVAS [11]. The eVAS also demonstrated excellent reliability, with regard to both individual ICC 0.90 (95% CI 0.82-0.95) and ICCa 0.97 (95% CI 0.95-1.0). Within a child and adolescent population, this study recognized the interchangeability of the eVAS and pVAS owing to moderate-to-good intermethod and intramethod reliabilities as determined by an ICC value of 0.80 (95% CI 0.70-0.87) and ICCa value of 0.80 (95% CI 0.69-0.87), respectively. Although this methodology has never been performed within this age range, Sánchez-Rodríguez et al reported that the mobile app “Painometer,” which includes a 100-mm VAS scale, was concordant with its traditional counterparts from ages 12 to 19 years [13].

There are multiple methodological strengths of this study. Specifically, as previously recommended by Escalona-Marfil et al, this trial included block randomization during data collection for the eVAS and pVAS allocation sequence and also introduced a 5-minute interval between measures to reduce possible pain recall bias. This study suggested that fingerprints might remain visible on the screen during the eVAS recordings [17]. The current Australian study prevented traceable fingerprints by wiping clean the tablet’s screen at the end of each recording. For the first time, this study reports the interchangeability of using eVAS compared with pVAS in pediatric participants from 10 years of age.

Occasionally, when using the traditional pVAS, patients may draw the line before the zero (0) or after the 100 point; therefore, these scores become invalid. Instead, the introduction of the eVAS app as part of regular pain evaluation prevents patients from recording their pain level outside the pVAS line, subsequently avoiding invalid scores.

For clinicians and researchers, especially those involved in community nursing or domiciliary visits, the time, cost, and space savings of data storage using the eVAS may be considerable when compared with the traditional pVAS, where manual transcription into clinical notes is required. The proposed eVAS app allows for automatic calculation of the VAS score, preventing possible human errors while using a ruler.

Patients may draw multiple lines on the pVAS or use inappropriate pens or thick highlighters. This can become confusing for clinicians to thoroughly interpret and record the intended results. A recent scoping review of systematic reviews highlighted that mHealth and hand-held electronic devices allow for accurate and complete medical documentation, providing instant access to reliable health data that may support clinical decision making [33]. Novel mHealth tools, such as the eVAS app, may make the work of health professionals even more efficient and increase reliability during their clinical assessments.

The eVAS app allows for the objective monitoring and recording of patients’ pain levels. This mHealth tool might become advantageous for those patients living in geographically remote areas, where limited access to specialists is apparent. Patients and parents/caregivers may not always be required to visit the hospital, consequently saving the time and money required to travel long distances from rural areas. If promptly introduced within different pediatric and adult pain clinic services, the eVAS may support early pain detection, preventing incidences of unnecessary prolonged pain, with a consequent improvement in the patient’s quality of life. This improved clinical management of pain may also lead to a reduction in absence from school or work.

Although not utilized within this study for data analysis, the eVAS app is capable of recording the time and day when the measures are taken. This important feature could be integrated within clinical settings and automatically reported within patient clinical records to highlight any diurnal variation in pain perception. Future trials could therefore also investigate the possible fluctuation of pain within a day. Notably, this may provide greater understanding of the complex nature of pain in response to environmental conditions or treatment plans [34].

This study further adds to the growing body of evidence that supports the use of digital technologies in health care. eHealth and mHealth have already been extensively used as tools for education, diagnosis, and management of pathologies such as diabetes [35,36], pediatric rheumatology [37], polycystic ovarian syndrome [38], and alcoholism [39]. At present, there are limited approaches available that combine evidence-based practice with health apps [40]. Portelli et al reported that most of the current apps available for pain management are rarely supported by an evidence base and may be misleading with their claims [41]. In addition, there are still limited regulations regarding data privacy for information collected from these apps [42]. Alarmingly, Blenner et al highlighted that many apps for diabetes management sold data to third parties without disclosure, even with a privacy policy stating that data were not going to be shared for commercial benefit [43]. This indicates the importance of further high-quality research into mHealth and eHealth regarding data privacy. More effort is also needed with regard to educating patients and practitioners in the use of apps that fully adhere to the guidelines clearly set by the WHO in evaluating digital health outcomes [18,44,45].

### Limitations

There are some limitations that should be considered while interpreting the findings of this study. First, despite a recruitment effort, a balanced number of children and adolescents (n=47, 43%) and adults (n=62, 57%) was not obtained. This was due to logistical school issues in obtaining signed consent forms from parents during busy school terms. The relatively smaller pediatric sample size may have had an impact on the overall ICC values obtained from the child and adolescent population, in comparison with the adult population. Second, it should be noted that data were collected in a convenient sample of people from the community who were not exposed to high levels of pressure pain with the Wagner Force Dial. Future studies may consider testing the eVAS with different intensities of pressure that would be deemed ethically acceptable. Third, a 1-minute interval is typically considered a suitable time gap to measure pain generated by pressure application [22,23]. During this trial, a 5-minute gap was adopted to record the pain generated by the pressure application (8.5 kg/cm^2^) on the participant’s thumb. The 5-minute gap was chosen to reduce any possible pain recall, especially among pediatric participants and to allow full recovery of sensory function of the thumb. There is possible anchoring bias on repetition of the two tests within a 5-minute interval. A 1-minute interval is deemed appropriate to assess a noxious stimulus without temporal summation [22,23,26]. Within the concept of pain measurement, recall bias may include a psychosocial aspect acknowledging that pain may be amplified or reduced after an extended period of time [46]. Additionally, pain perception within a population may fluctuate day to day [47]. However, for the purpose of this cross-sectional study and for determining interchangeability between the pVAS and eVAS, it is imperative that a single controlled stimulus is used and that measurements are undertaken within the same environment to eliminate confounding. Finally, although the child and adolescent population had slightly lower reliability relative to the adult population, this evidence supports the use of the eVAS in the pediatric population. A possible cause for the lower reliability relative to the adult population is the difference in scale length across platforms. The eVAS line width was 13.5 cm compared with 10 cm in the pVAS. Conceptual understanding of scales may differ between adult, and child and adolescent groups [48]. To encourage consistent conceptual understanding of the study in a large age range (10-75 years), the participants were made aware of the difference in sizes of the scales and asked to mark in a ratio. It is plausible that owing to the possible limited understanding of spatial ratio, there might be an impact on the results from younger participants [48].

In conclusion, the use of technology by children, adolescents, and adults is growing and is evident across multiple settings [49,50]. This study highlights the need for further investigation regarding the transferability of an eVAS pain app to different smartphone and tablet screen sizes that are already largely accessible within the community.

### Clinical Implications

Monitoring and evaluating digital health interventions can be challenging, but have become requirements within the mHealth and eHealth fields [18]. This study specifically supports the adoption of these easy-to-use and validated pain assessment mHealth methods that have excellent reliability in adults and moderate-to-good reliability in children and adolescents. The emerging field of digital health presents an evolving cultural shift within health care settings. The growing use of digital mHealth has the potential to improve pain management. eHealth and mHealth have the ability to improve adherence to pain reporting [51,52], allow real-time data capture [53], and improve communication between practitioners and patients [54].

### Conclusion

This study provides supporting evidence on the interchangeability of the eVAS and pVAS in child and adolescent, and adult populations. The introduction of similar validated eVAS pain apps may greatly increase the quality of reliable data accessible to clinicians, thereby improving the well-being of symptomatic patients. Most importantly, the use of mHealth in pain management may also facilitate timely clinical decisions, improve patients’ self-management and overall awareness in the progression of their pain levels, and become an integrated approach consistent with the eHealth goals of the WHO and Australian Health Authorities [18,19]. Further research is needed on the use of these pain apps among symptomatic children, adolescents, and adults to ascertain the possible impacts of this new technology in these populations [55,56].

## Abbreviations

AHP: allied health professional
eVAS: electronic visual analog scale
pVAS: paper visual analog scale
VAS: visual analog scale
WHO: World Health Organization
